# Pushing the Pace of Tree Species Migration

**DOI:** 10.1371/journal.pone.0105380

**Published:** 2014-08-27

**Authors:** Eli D. Lazarus, Brian J. McGill

**Affiliations:** 1 Environmental Dynamics Lab, Earth Surface Processes Group, School of Earth and Ocean Sciences, Cardiff University, Cardiff, Wales, United Kingdom; 2 School of Biology and Ecology, University of Maine, Orono, Maine, United States of America; The Ohio State University, United States of America

## Abstract

Plants and animals have responded to past climate changes by migrating with habitable environments, sometimes shifting the boundaries of their geographic ranges by tens of kilometers per year or more. Species migrating in response to present climate conditions, however, must contend with landscapes fragmented by anthropogenic disturbance. We consider this problem in the context of wind-dispersed tree species. Mechanisms of long-distance seed dispersal make these species capable of rapid migration rates. Models of species-front migration suggest that even tree species with the capacity for long-distance dispersal will be unable to keep pace with future spatial changes in temperature gradients, exclusive of habitat fragmentation effects. Here we present a numerical model that captures the salient dynamics of migration by long-distance dispersal for a generic tree species. We then use the model to explore the possible effects of assisted colonization within a fragmented landscape under a simulated tree-planting scheme. Our results suggest that an assisted-colonization program could accelerate species-front migration rates enough to match the speed of climate change, but such a program would involve an environmental-sustainability intervention at a massive scale.

## Introduction

Plants and animals have responded to past climatic changes by shifting the spatial boundaries of their geographic ranges. Such migratory responses to climate change have proved remarkably successful for many species. For example, in North America, no species of small mammal [1] and only one species of tree [2] went extinct in the dozen-plus glacial cycles over the Quaternary. With the approximately 7°C warming that occurred 20–10 kya (punctuated by rapid rates of temperature change over shorter intervals within that period), plant and animal ranges in North America shifted up to the order of 1000 km [3]–[5]. Pollen records show evidence that temperate tree species migrated at rapid rates (100–1000 m yr^−1^) following the last Ice Age [6]. Warming of approximately 3.5°C is expected over the next 100 years [7]. As a first approximation, linearly extrapolating the ratio of warming (7°C) to range adjustment in the early Holocene (1000 km) suggests that ranges could shift an average of 500 km in the coming century (5 km yr^−1^) [8]. Reconciling paleo-rates with observed spreading patterns of plant taxa has motivated a wealth of ecology research into mechanisms for rapid migration, such as long-distance dispersal [9], [10]. However, paleo-range shifts occurred in the absence of anthropogenically dominated landscapes. How human uses of otherwise suitable habitat will affect climatically driven range migration across landscapes is an open, fundamental question in environmental sustainability. Here we explore this question in terms of temperate-forest tree species.

Field measurements of typical seed dispersal distances would suggest that tree fronts migrate across a landscape by a process of local diffusion, at rates significantly slower than the velocities reflected in pollen data [9]. But seeds are occasionally carried long distances from their source by wind or by animals [11]. If those seeds mature into trees that in turn dispense seeds, the plant species may migrate at rates that far exceed diffusive propagation [6], [9]. Existing models of tree migration by long-distance dispersal produce migration rates between approximately 100–200 m yr^−1^ [9], [10], [12]. A global analysis of temperature-change rates across geographic gradients and biomes finds that temperate broad-leaf and mixed forests, which includes the North American taxa that spread by wind-blown dispersal [10], will need to shift at a mean velocity of 350 m yr^−1^ [13]. These required migration rates appear to exceed the fastest modeled rates, but may fall within the ranges empirically observed in the last deglaciation. Migration rates barely sufficient to track with climate, combined with the well-documented effect that landscape fragmentation further impedes migration [14]–[19], points to the apparently unequivocal conclusion that climatic change will outpace the migration of wind-dispersed tree species through human-dominated landscapes. One of the few known cases in which climate-driven species migration was impeded comes from Europe, where east-west mountain ranges and the Mediterranean Sea prevented trees and plants from advancing far enough south during Pleistocene glaciation, resulting in a high proportion of extinction [20]. By extension, understanding how human fragmented landscapes interfere with migration rates might mean the difference between minimal extinction rates and massive extinction rates in next few hundred years.

Assisted migration, assisted colonization, and species translocation are already common conservation practices applied to variety of plant and animal species [21], [22]. Unwitting or unintended human introduction of alien, invasive species to new environs via long-distance transport pathways is arguably a version of the same idea. Assuming disparity between rates of climate change and temperate-forest migration is an inherent condition of the future sets up a new question: how much human intervention might windblown-dispersal taxa need to keep pace with climate change? Although assisted migration remains controversial [23], here we neither advocate for nor reject it. We simply ask: if humans were to deliberately augment the migration rates of temperate-forest tree species, what would the scale of that intervention need to be relative to the natural dynamics driving the migration front?

**Figure 1:**
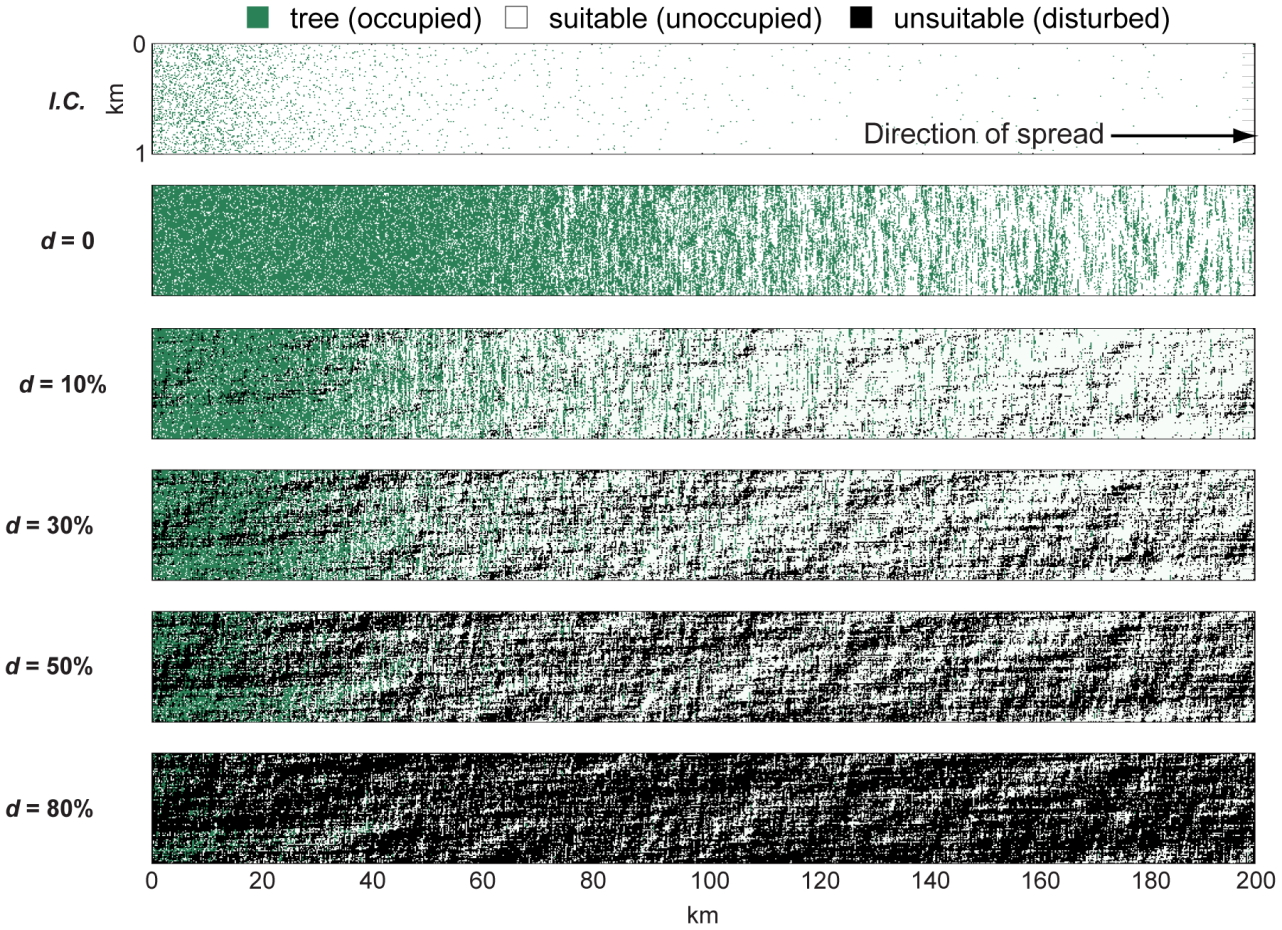
Representative model domains. Top panel shows the model's initial condition (*I.C.*) for tree positions in an undisturbed landscape. Subsequent panels show the resulting domains after 1000 years given different extents of disturbance (*d*), where disturbance is spatially autocorrelated (Hurst exponent *H* = 0.99). Apparent lineations in the ordinate dimension are a distortion in the aspect ratio, not an artifact of the model.

Here we present results from a probabilistic spatial model that simulates the passage of a generic, wind-dispersed tree species through a two-state, cellular landscape in which cells are either suitable or unsuitable for trees (Fig. 1 and fig. S1; additional model explanation, parameters, and source code are provided in File S1; data shown in figures are provided in File S2.). Carefully abstracted models that capture the salient dynamics of well-defined systems are useful tools for insight into how natural systems may respond to changes in forcing, and by design are more exploratory than explicitly predictive [24], [25]. Cellular architecture has been used in several idealized spatial models of migration patterns through heterogeneous habitats and fragmented landscapes [16], [26]–[28]. We use our model to test how augmenting the number of successful seedlings accelerates the rate of tree front migration. Our results suggest that relatively small changes in the annual number of successful seedlings could have a comparatively large effect on biome migration rates.

## Model Design

### Spatial domain

Our model operates on a gridded domain with a width-to-length ratio of 1∶200 km and a periodic (i.e. wrap-around) boundary in the width dimension (fig. S1). The periodic boundary condition makes the domain infinitely wide; the extended length dimension allows a fixed aerial view (200 km) large enough to frame potentially rapid incoming migration fronts (> km yr ^−1^). Each grid cell is 10×10 m, and can accommodate a single tree. Although these cell dimensions are coarse for small, young trees, they are consistent with crown widths of large trees measured via remote sensing in a North American temperate mixed forest [29]. We run the model for 1000 years so that initial conditions dissipate and migration dynamics may play out in full (e.g. until available area is fully populated, or all trees die out).

### Seed dispersal

**Figure 2:**
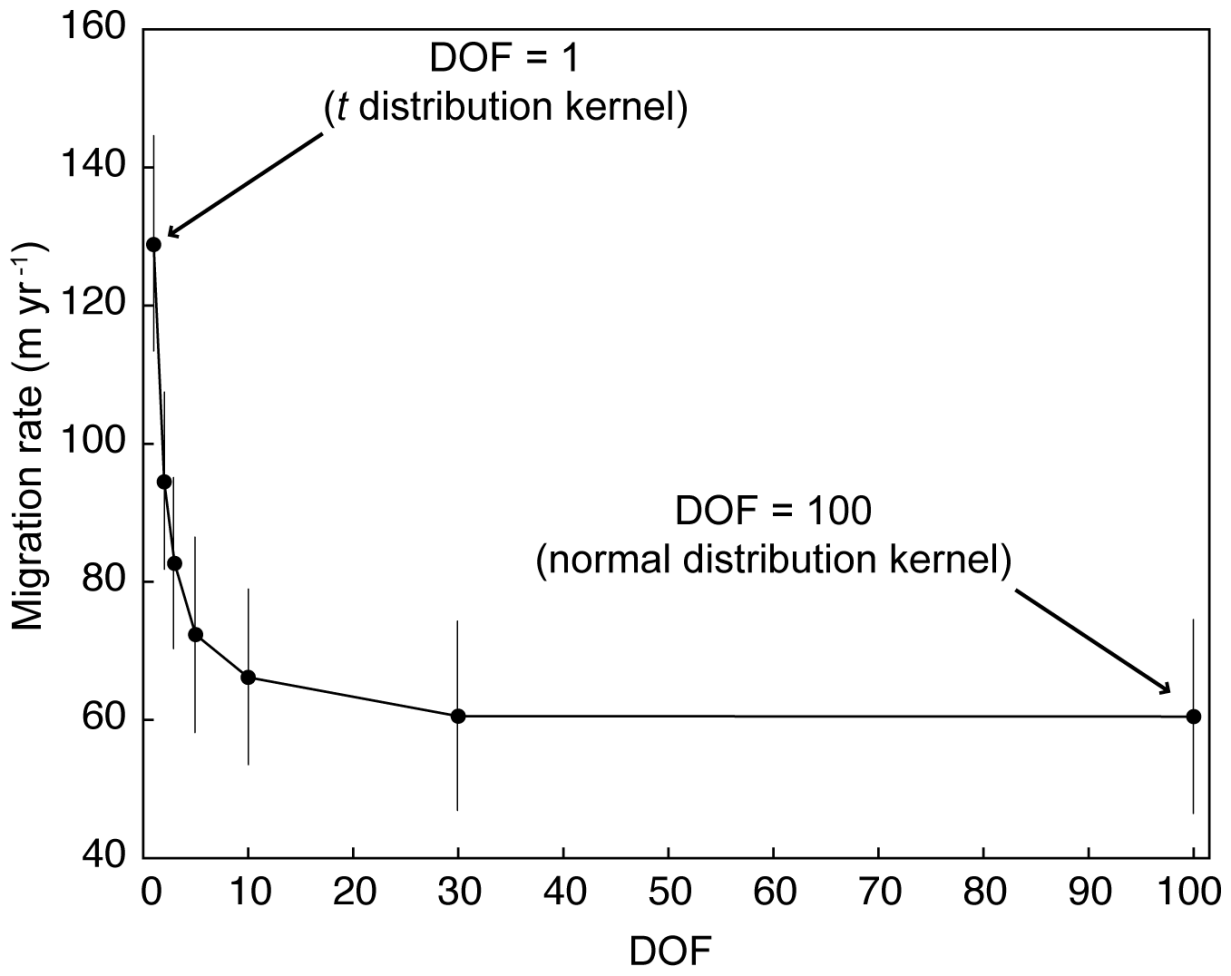
Mean migration rates with increasing degrees of freedom (DOF) in a *t*-distribution kernel for long-distance dispersal. Bars indicate envelope of one standard deviation. When DOF = 1, the dispersal kernel is "heavy tailed." When DOF is >>1 (e.g. DOF = 100), the dispersal kernel has the comparatively limited range of a Gaussian distribution, and resulting mean migration rates represent the rate of spreading by local diffusion.

Accounting for infrequent long-distance dispersal events requires a statistical function with a "heavy tail," so that the maximum dispersal distance may be very large even if the average dispersal distance is short. Other models have used various heavy-tailed probability distribution functions to drive dispersal distance [9], [10], [12], [16], [30]–[32]. Seed dispersal distance in our model is generated by a *t*-distribution with a degrees-of-freedom variable that changes the "heaviness" of the distribution's tail: the minimum degrees of freedom (DOF = 1) allows the longest dispersal distances; by comparison, a high degree of freedom (e.g. DOF = 100) approximates a Gaussian distribution, with a dispersal pattern limited to local diffusion (Fig. 2). We use the *t*-distribution because of the straightforward means by which the distribution can be shifted from Gaussian to heavy tailed for comparative scenarios. In the results presented here, the initial model domain (Fig. 1) is populated with trees according to a *t*-distribution (with DOF = 1) centered on the far left edge.

### Tree characteristics

Each tree in our model is assigned biological characteristics for tree fecundity (seeds tree^−1^ yr^−1^, as a function of tree age), seed dispersal distance, and seedling survival according to probability-distribution functions constrained by empirically derived parameters for North American wind-dispersed trees (see Table S1) [10]. Maturation age (yr) and seed-crop interval (yr) are sampled from normal probability distributions. Longevity (yr) is sampled from a gamma distribution, which allows a few long-lived trees to persist among a community of trees whose average age is younger. Each model tree broadcasts some number of "successful" seeds (seeds that will germinate into seedlings) every few years as a function of its maturation age, seed-crop interval, and fecundity. We treat tree fecundity as an increasing parabolic function of age; the number of viable seedlings per tree is then a percentage of that fecundity, determined by a probability of seedling survival for a given tree's seed crop in a given model year. Each seed is assigned a randomly selected compass direction and a dispersal distance that corresponds to a grid cell in the domain, which, if unoccupied, becomes a new tree with parametric values resampled from the probability-distribution functions for biological characteristics.

### Landscape disturbance

We create disturbance patterns by first generating a domain-scale layer of random values between 0–1 that are either highly spatially autocorrelated (e.g. high values occur near other high values, and likewise for low values) or are spatially uncorrelated (spatially random), with the degree of correlation controlled by the Hurst exponent [17], [19], [33]. We use Hurst exponents of *H* = 0.99 and *H* = 0 for our correlated and uncorrelated landscapes, respectively. We control the percentage of landscape disturbance by isolating cells in the grid that exceed a given threshold value between 0–1; cells whose values exceed the threshold are rendered "disturbed" and assigned a value  = 1; all other cells (undisturbed) are set  = 0. For comparability across scenarios, the underlying Hurst-model landscape (for a given value of *H*) is kept constant. The threshold changes, and therefore the overall pattern of disturbance, but retaining the same underlying Hurst model means that a patch disturbed at a high threshold (yielding a low percentage of disturbance) will persist at lower thresholds. Furthermore, the disturbance pattern is held constant through time within a model run. Trees may occupy any undisturbed cells in the domain; seedlings distributed to disturbed cells are removed.

### Assisted colonization

Assisted colonization occurs in the model when new trees are "planted" at the end of a model year, after all existing, active trees in the domain have broadcast their seeds (fig. S1). Grid cells in which new trees are planted are randomly selected from among all undisturbed cells not already occupied by an existing tree. We do not account for ways in which proximal areas of disturbance might impinge upon tree health, or disturbance "edge effects" [34], [35]: planted trees are governed by the same parameters as trees growing "naturally" in the model.

The number of trees planted is based on the average number of new trees that grow in the model when (a) there is no landscape disturbance and (b) dispersal distances are generated by a heavy-tailed *t*-distribution function (DOF = 1). In the results shown here, that average number is 2000 trees yr^-1^. These conditions constitute the model's the ecological baseline. Assistance is thus a function of increasing the fixed number of new trees per year by a percentage *K*.

Planting in the model is not explicitly organized in the direction of migration: available sites at the distal edge of the domain are not prioritized over other available sites. However, because the distal edge of the initial model domain is comparatively empty, distal planting sites are more numerous and therefore more likely to be selected from among the sites available, especially when the rate of assistance is high.

## Results

When the entire model domain is hospitable to seedling growth, heavy-tailed dispersal distances drive front migration rates of approximately 130 m yr^−1^ that are nearly double those driven by diffusive dispersal (Fig. 2). Front migration rates in this model, measured as the change in mean tree position across the domain, are comparable to rates reported for other, analogous long-distance dispersal models [9], [12], including the study from which our ecological parameters derive [10]. This agreement in rates is not a foregone conclusion. Although the independent biological variables in the model are constrained by parametric distributions that are similar to (but not the same as) those used in other studies, migration rate is an implicit variable that arises from the collective state of the full model domain over time. The agreement we find provides a good validation of our modeling approach.

**Figure 3:**
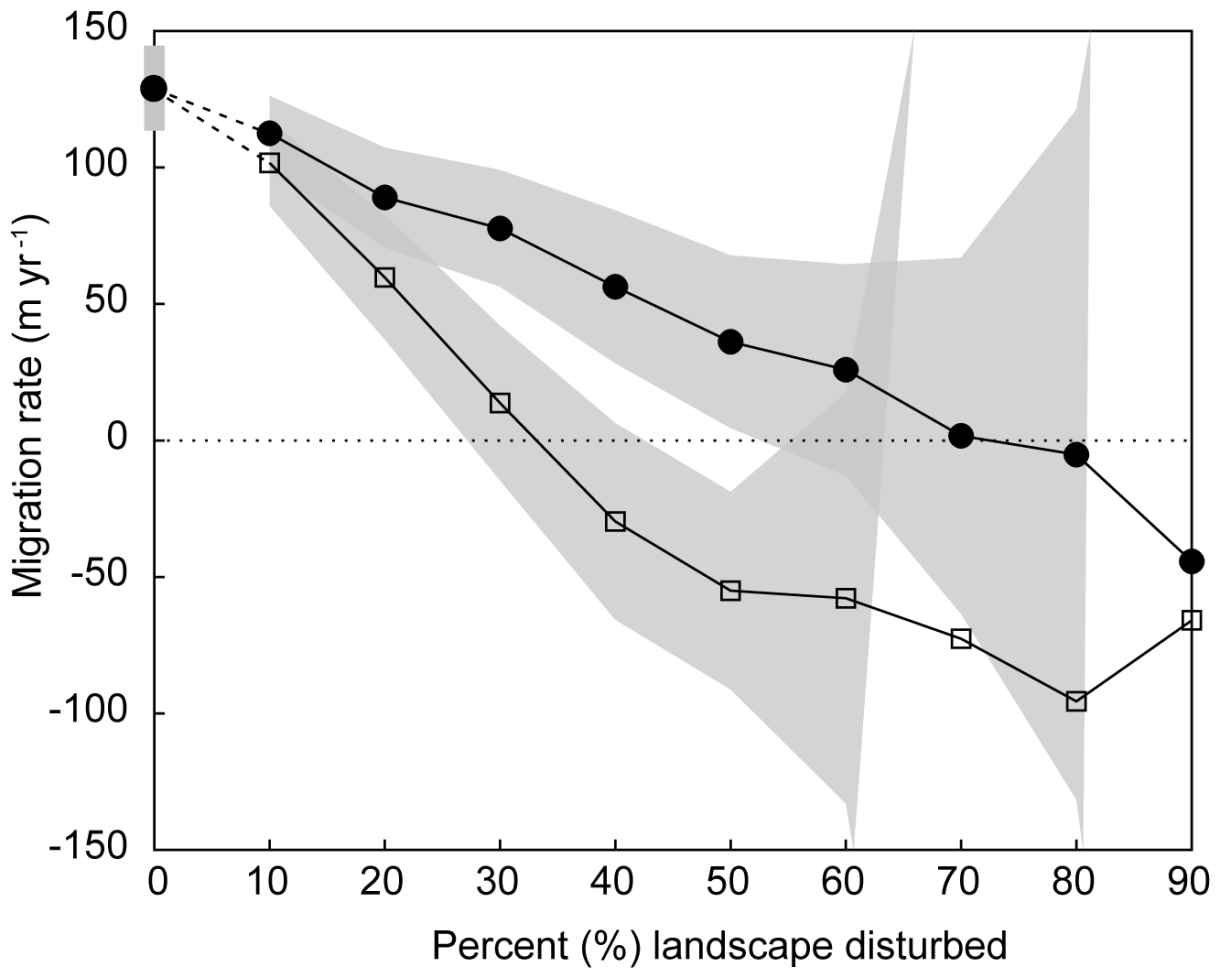
Mean migration rates (via long-distance dispersal, given *t*-distribution kernel with DOF = 1) through increasing proportions of landscape disturbance when disturbance is spatially autocorrelated (black dots; *H* = 0.99) and spatially random (open squares; *H* = 0). Gray regions denote envelope of one standard deviation. Negative values indicate conditions under which the tree front receded from the domain (died back) rather than advanced across it.

Fragmenting the model domain with landscape disturbance shows that migration rate and disturbance are negatively correlated (Fig. 3), as other disturbance models have also shown [16], [17], [19]. Furthermore, the presence or absence of pattern in the disturbance regime affects the migration rate. When disturbance across the model landscape is spatially random (*H* = 0), the migration front ceases to advance once the total area disturbed reaches 40%. This propagation result grants some comparison to percolation models for square lattices. The percolation threshold for a square lattice is approximately 59% [36], meaning a fully connected route through the lattice exists as long as disturbance to the lattice is less than 41%. Long-distance dispersal in model does not necessarily succeed where random walks cannot: even a species capable of dispersal between non-adjacent sites does not advance through a randomly fragmented domain (*H* = 0) in which disturbance exceeds the percolation threshold. When disturbed areas are highly autocorrelated in the model (*H* = 0.99), leaving contiguous reaches of potential habitat (corridors) relatively intact, migration rates decrease more slowly, tolerating up to 70% total disturbance (Fig. 3). Most importantly, none of these migration rates—including the maximum rates in our idealized, undisturbed scenario (Fig. 3)—come close to matching the 350 m yr^−1^ "velocity of climate change" modeled for temperate broad-leaf and mixed forests [13].

**Figure 4:**
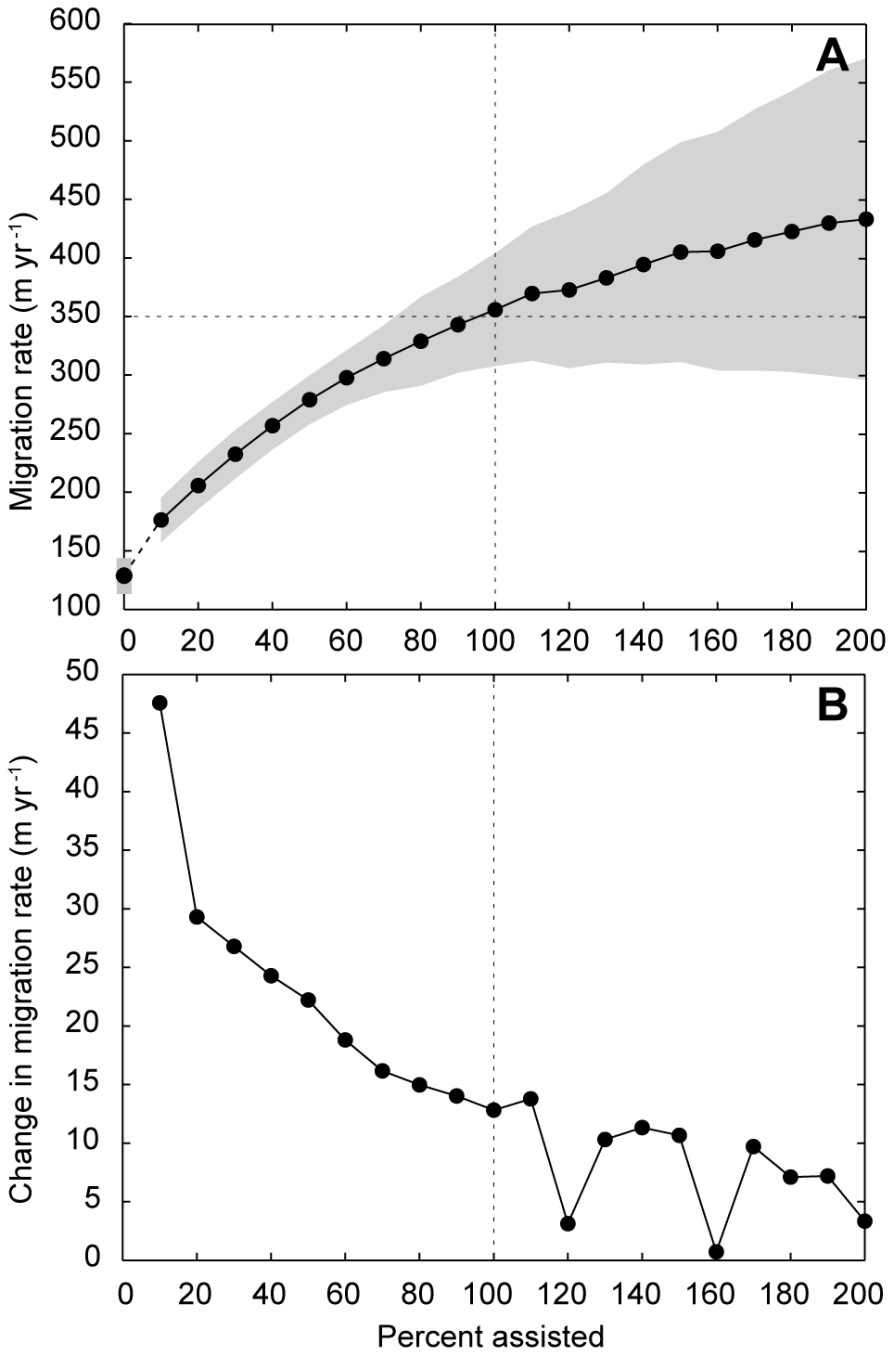
Mean migration rates with assistance. (A) Mean migration rate through an undisturbed landscape with increasing levels of assisted colonization. Gray regions denote envelope of one standard deviation. When the number of naturally successful seedlings is doubled (*K* = 100%), migration rates become comparable to the measured mean velocity of range shift for temperate deciduous forests [13]. (B) First derivative of migration rates plotted in (A), showing that increasing assistance has a diminishing return on migration-rate acceleration, especially for *K*>100%.

That apparent lag motivates our simplified version of assisted colonization to accelerate migration rates. The key assumption we make is that all of the trees deliberately introduced to the domain can grow to be viable adults, subject to the probabilistic limits of tree longevity and fecundity imposed in the model. To consider the effect of assisted colonization on rates of tree front migration through an undisturbed landscape (Fig. 4), we systematically increase the number of planted trees by *K* = 10–200% in increments of 10% (e.g. when *K* = 10%, 200 trees are planted annually). Planting begins in model year  = 200 and continues until year  = 1000. For these experiments, we calculate mean migration rate over the first hundred years of assisted colonization (model years 201–300); after 100 years of planting, the migration front effectively halts as available spaces in the model domain fill up (fig. S2).

**Figure 5:**
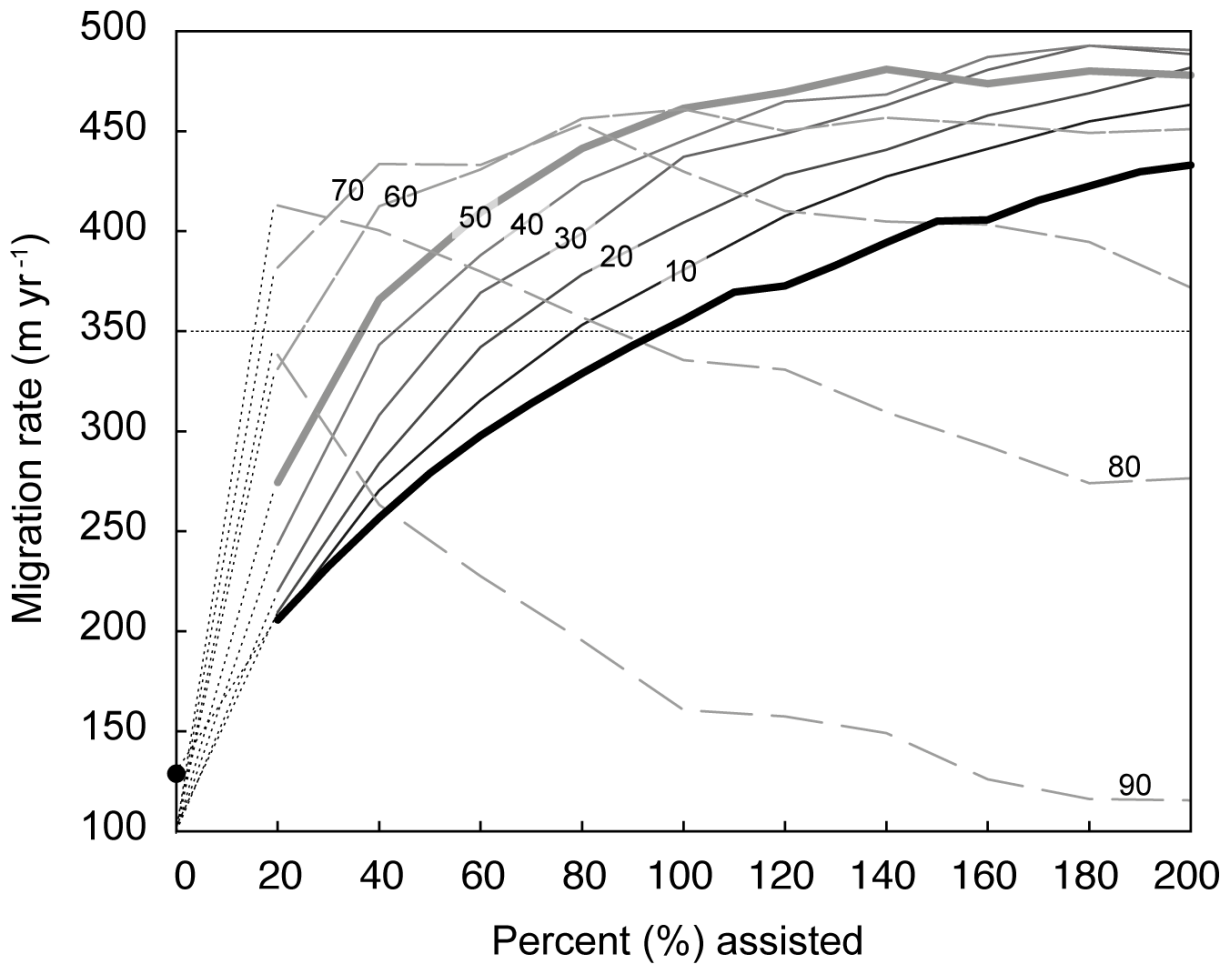
Assisted migration rates through landscapes with increasing levels of disturbance (lines labeled 10–90%), where disturbance is spatially autocorrelated (*H* = 0.99). Bold line shows assisted migration rates through an undisturbed landscape. Even small amounts of assisted colonization have a strong effect on migration rates as landscape disturbance increases.

These simulations suggest that doubling the number of annually successful trees (*K* = 100%) in an undisturbed landscape would result in migration rates equal to the necessary mean velocity of range shift of 350 m yr^−1^. We also find that even the weakly organized strategy of assisted colonization in this model has a pronounced, accelerating effect on migration rates in fragmented landscapes where disturbance is highly autocorrelated (*H* = 0.99) (Fig. 5). This result is not necessarily surprising. Unlike stochastic seed dispersal as a means of colonizing undisturbed areas, planting ensures that trees occupy available space. In a highly disturbed landscape, planting makes colonization a comparatively targeted process, which only makes migration rates faster. However, a caveat associated with determining the migration rate within a disturbed domain is that the calculation is based on fewer trees overall than are present in an undisturbed domain. A high migration rate can appear to be maintained through large extents of disturbance with less intervening assistance (Fig. 5), but the total population of trees is smaller.

## Discussion

### Model uncertainty

Migration rates in the model depend principally on the presence or absence of a heavy tail in the distribution of dispersal distances (Fig. 2), and on the empirical constraints on the probability-distribution functions [10]. We ran our model using other heavy-tailed probability-distribution functions for dispersal distances (e.g. power-law functions) constrained by the same statistical ranges and found that, although the resulting landscapes differed in detail, the model produced comparable migration rates with similar migration-front dynamics.

Given that our interest is in the migration rate of a generic pattern at the domain scale, our model is more phenomenological than mechanistic and shares the same parametric sensitivities as others of its kind [32]. The spatial distribution of surviving seedlings, not just the distribution of dispersal distances, is an important component of migration, but here we do not account for potential differences in seedling success (e.g., germination, competition, survivorship, seed production) with distance from the parent tree [32], [37].

Furthermore, although climate change motivates our interest in modeling tree-species migration through fragmented landscapes, we do not attempt to model climate-change effects on ecological traits [38] or on habitat traits such as soil conditions [39]. To keep our model focused on dispersal, we treat climate change here as the exogenous driver of the minimally required rate of migration needed to avoid extinction. Assuming that the trees in this model migrate into and across a landscape already ecologically suitable for them to inhabit emphasizes the relative effects of disturbance and assistance on migration dynamics. We thus treat climate change as an implicit migration-rate threshold: even in this "fastest-case" scenario, for a given pair of disturbance and assistance conditions, are migration rates faster or slower than the projected rate they must achieve to survive?

Of course, the inherent uncertainty in our model derives far less from tree ecology than from patterns of landscape disturbance and the application of assisted migration, the abstracted human activities we represent. As Higgins et al. [32] remark in their review of plant-migration forecasting, "inherent uncertainty cannot be reduced by improving model or parameter uncertainty." By design, our model is a suitable tool for reasoning by analysis rather than by analogy [40]: a means of exploring inherent uncertainty through the encapsulation of relevant dynamics rather than in a scaled simulacrum of the real world.

### Landscape corridors and assisted migration

Protecting contiguous reaches of undisturbed habitat by concentrating new disturbance near existing disturbed areas is the premise behind ecological-corridor conservation practices [23], [41], [42]. Some researchers have discussed the extent to which the mechanism of long-distance dispersal could enable a tree species, in the absence of an uninterrupted habitat corridor, to migrate along a series of proximal habitat "islands" [16], [17]. Our results suggest that even when available habitat is strongly autocorrelated, once half the landscape domain has been disturbed, long-distance dispersal advances the front no faster than diffusive passage through an undisturbed landscape (Figs. 2 and 3). Moreover, beyond approximately 70% disturbance, the migration front fails to advance even with long-distance dispersal and in the presence of autocorrelated habitat islands (Fig. 3).

Indeed, if migration fronts effectively halt with disturbances exceeding 70% (Fig. 3), then migration by assisted colonization might be the only means by which a front of trees could move through a landscape so heavily disturbed. Moreover, such planting and could accelerate migration rates within decades (fig. S2), commensurate with time scales relevant to climate-change projections and mitigation actions [7]. We find that migration rates in our model increase with increased assistance, but augmenting the number of annually successful trees by more than 60–80% has a diminishing effect on acceleration (Fig. 4). Within a fixed domain (e.g. a spatial domain that fills as the migration front advances, rather than tracking with the front) an economy of scale therefore may govern the rate-accelerating effects of assisted colonization. Assuming tree planting has an associated fixed cost (i.e. each tree requires time and labor to be planted; trees for planting need to be raised in nurseries, etc.), at least in the context of a coordinated, top-down climate-adaptation program [43], [44], this diminishing advantage in the relative effect of planting more trees could manifest in both ecological and practical terms.

Representing a tree-planting strategy as a stochastic, opportunistic process is at least as reasonable as our stylized treatment of landscape fragmentation, and likewise is as reasonable as assuming that in a real tree-planting campaign every site available ecologically would be available in practice. Targeting planting efforts at the far edge of a species' geographic range is one way to accelerate migration, but trees planted beyond the advancing front are not the only ones capable of affecting the migration rate. A consequence of long-distance dispersal is that even a tree planted behind the migrating front is capable of broadcasting a large number of viable seeds toward the distal edges of the geographic range. Perhaps the primary benefit of assisting tree-species colonization and migration, as opposed to managing animal species that roam, is the ability to selectively and specifically establish sensitive taxa in undisturbed environs irrespective of fragmentation intensity.

### Implications

The lag between tree migration rates and the velocities of their preferred environments raises interesting implications for ecological dynamics at ecotonal boundaries. For example, temperate-broadleaf and mixed-forest biome environments in North America will need to move to higher latitudes at an average rate of 350 m yr^−1^ to track with climate, but boreal forest and taiga environments, also moving northward, are shifting at a faster average of 430 m yr^−1^ [13]. Ecotones between biomes demarcate zones where individuals are living at the fringe of their environmental tolerances and are arguably the most sensitive to relative differences in surface-temperature velocities [45]. Habitat fragmentation in a shifting ecotonal zone is therefore especially complicated and problematic [45], [46]. Conceivably, habitat fragmentation across an ecotone could result in one principal biome departing before the next can establish, making the liminal landscape a kind of anthropogenic biome with its own ecological signature [46].

An intentional planting program therefore represents a compromise: a direct intervention that enables an erstwhile natural biome to persist under anthropogenic pressure. Our model results suggest that this would have to occur on a massive scale to be effective for a continental ecotone. Targeted plantings at specific locations assumes a top-down approach to climate-change adaptation, but such adaptation is at least as likely to occur—and is perhaps more realistic—through a bottom-up approach in which people act locally (i.e., plant trees in their neighborhood) [43], [44]. An environmental conservation campaign resembling the simplified one in our model—a tree-planting campaign without explicit spatial targets but enacted at a large enough scale to potentially accelerate the migration rates of a forest biome—is not without precedent. The Green Belt Movement, founded by the late Nobel Laureate Prof. Wangari Maathai, is credited with having planted more than 51 million trees in Kenya since 1977 through grass-roots community organization and mobilization [47]. The Billion Tree Campaign, a United Nations Environment Programme initiative that Maathai also championed, has registered over 12 billion trees planted worldwide since 2006 [48]. Given that landscape fragmentation and land-use changes will only further hinder slow-moving tree fronts as they follow fast-moving climate change, adding various common tree types to the list of migrating species that large-scale and local conservation organizations actively assist is both a necessary action and an environmental sustainability problem with, it would seem, a viable solution.

## Supporting Information

Figure S1 Schematics of our tree migration model: (A) representative initial condition without disturbance (arrows indicate how the periodic boundary operates on seed dispersal); final landscapes (B) without disturbance and (C) with disturbance; (D) sequence in a model year in which (D2) new trees are added by natural dispersal and (D3) by assisted colonization, resulting in (D4) the final landscape as shown. Actual model outputs are shown in Fig. 1.
(TIF)

Figure S2 Plot of mean front position versus time for increasing levels of assisted migration in the absence of landscape disturbance. Black line shows the natural, background migration rate (∼130 m yr^−1^), given a *t*-distribution dispersal kernel with DOF = 1. In this baseline scenario, approximately 2000 new trees grow in the domain per year. Blue lines show the effects of assisted migration regimes in which an additional *K*% trees per year are "planted", for *K* = 10–200%. Bold blue lines denote 50% increments; *K* = 200% is shown in red.
(TIF)

File S1 Document file containing model source code and explanatory text.
(DOCX)

File S2 Spreadsheet file containing data shown in the published figures.
(XLSX)
