# Supplementary figures and images for: Pushing the Pace of Tree Species Migration
Source: PLoS One. 2014 Aug 27;9(8):e105380. doi: 10.1371/journal.pone.0105380 (PMC4146538; doi:10.1371/journal.pone.0105380)

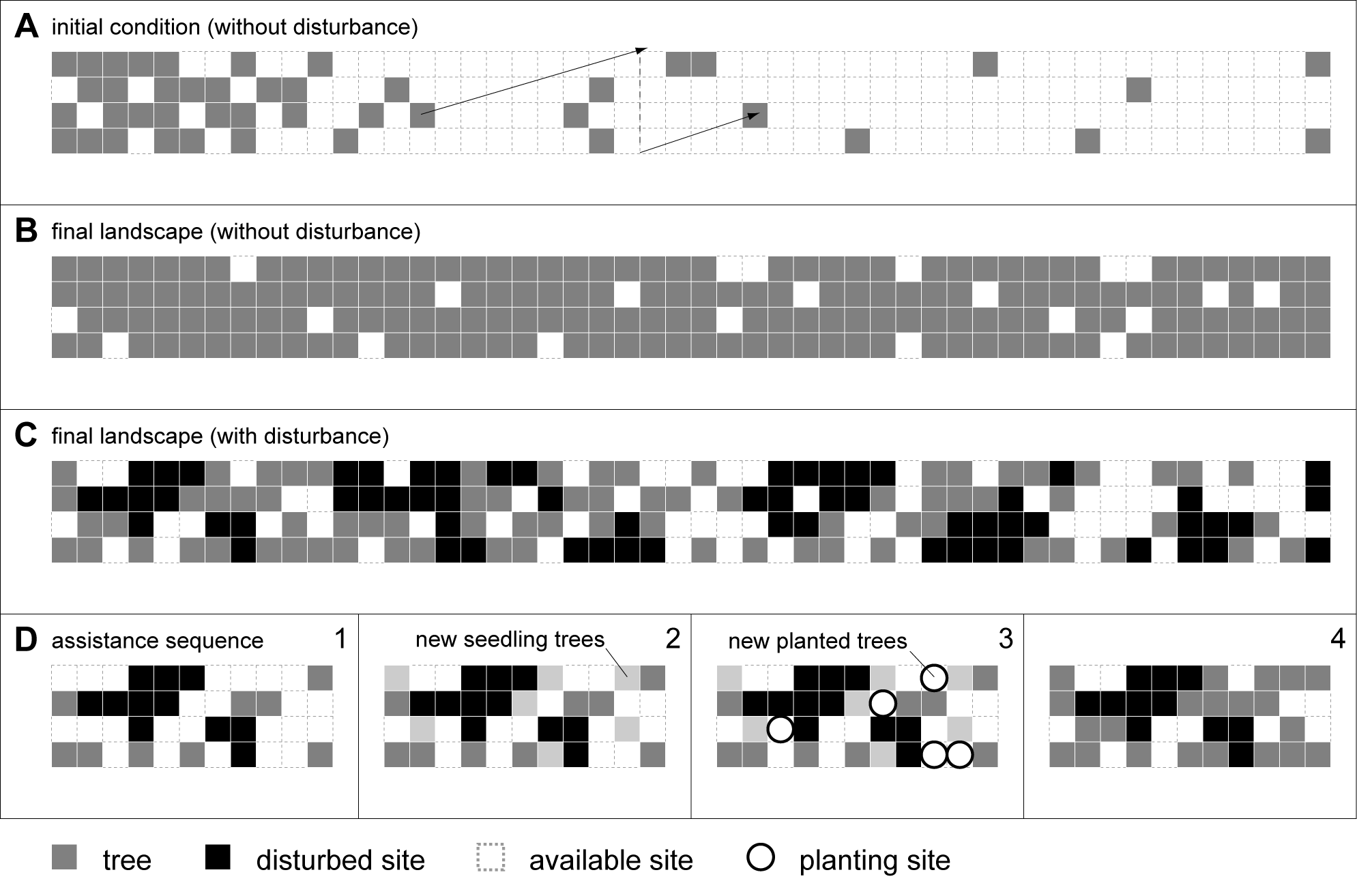

Supplement: Figure S1 — Schematics of our tree migration model: (A) representative initial condition without disturbance (arrows indicate how the periodic boundary operates on seed dispersal); final landscapes (B) without disturbance and (C) with disturbance; (D) sequence in a model year in which (D2) new trees are added by natural dispersal and (D3) by assisted colonization, resulting in (D4) the final landscape as shown. Actual model outputs are shown in Fig. 1. (TIF) [file pone.0105380.s001.tif]

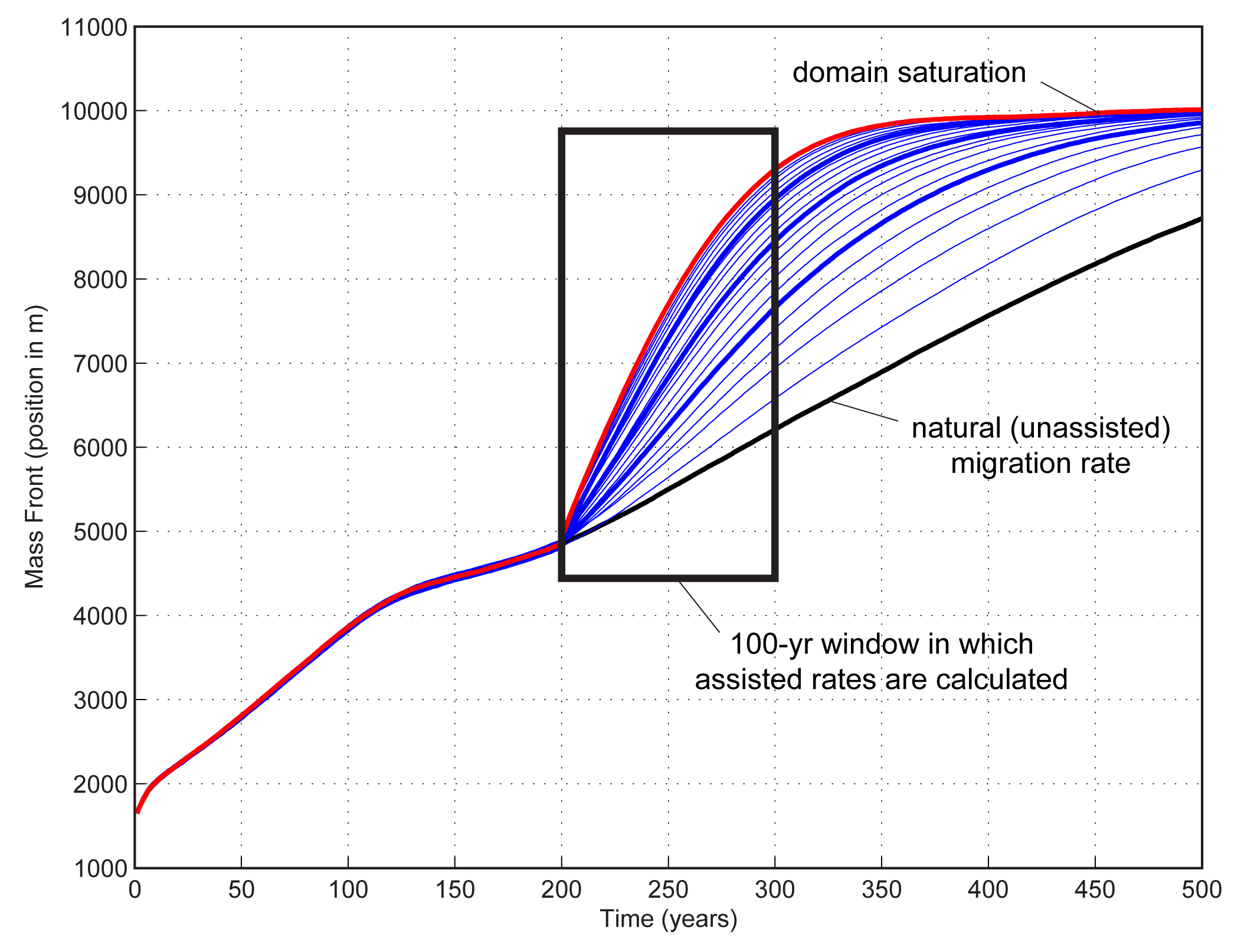

Supplement: Figure S2 — Plot of mean front position versus time for increasing levels of assisted migration in the absence of landscape disturbance. Black line shows the natural, background migration rate (∼130 m yr−1), given a t-distribution dispersal kernel with DOF = 1. In this baseline scenario, approximately 2000 new trees grow in the domain per year. Blue lines show the effects of assisted migration regimes in which an additional K% trees per year are "planted", for K = 10–200%. Bold blue lines denote 50% increments; K = 200% is shown in red. (TIF) [file pone.0105380.s002.tif]
