# Supplementary material for: Pushing the Pace of Tree Species Migration
Source: PLoS One. 2014 Aug 27;9(8):e105380. doi: 10.1371/journal.pone.0105380 (PMC4146538; doi:10.1371/journal.pone.0105380)
Supplement: File S1 — Document file containing model source code and explanatory text. (DOCX) [file pone.0105380.s003.docx]

**SUPPLEMENTARY INFORMATION**

*Title:* **Pushing the pace of tree species migration**

*Authors:* Eli D. Lazarus^1*^, Brian J. McGill^2^

*Affiliations:* ^1^Environmental Dynamics Lab, Earth Surface Processes Group, School of Earth & Ocean Sciences, Cardiff University, Main Building, Park Place, Cardiff, CF10 3AT, UK

^2^School of Biology and Ecology, University of Maine, 202 Deering Hall, Orono, ME 04469 USA

**Correspondence:* Dr. Eli Lazarus ([LazarusED@cf.ac.uk](mailto:LazarusED@cf.ac.uk))

*Keywords:* assisted migration, anthropogenic biomes, ecotones, environmental geography, biogeography, sustainability science

**Model code**

The following sections present the source code behind our model of front migration (and assisted migration) for a generic wind-dispersed tree species. The code here is written for Matlab, but can be amended for other languages as necessary.

Sections are as follows: (1) routine to generate the initial ecological conditions; (2) routine to generate Hurst-exponent landscapes for disturbance scenarios; (3) main model code; and (4) routine to plot the results presented in this paper.

Data for the published figures is available in a separate Excel file (also included in our Supplementary Information).

**Table S1.** Key model parameters.

|  | **Parameter** | **Range / Value** | **Formulation / Matlab Function / Notes** |
| --- | --- | --- | --- |
|  | *domain dimensions* | *r* = 10^2^; *c* = 2 · 10^4^ grid cells | *r* = rows; *c* = columns |
|  | *grid cell size* | equivalent to 10 x 10 m | one "tree" per grid cell |
|  |  |  |  |
| • | *maximum tree age* | 450 years | - |
| • | *seeds* | 300 – 4.5x10^5^ seeds tree^-1^ year^-1^ | function of age = 300 + 2 · (tree age)^2^ |
| • | *maturation age* | 3 – 60 years | uniform PDF on open interval (3, 60) |
| • | *intercrop interval* | 1 – 8 years | uniform PDF on open interval (1, 8) |
| • | *longevity* | 70 – 450 years | gamma PDF given by: 18 · *gamrnd*(5, 1, *W*, *L*) |
| • | *survival rate* | 10^-5^ – 0.02 | uniform PDF on open interval (10^-5^, 0.02) |
|  |  |  |  |
|  | *angle* | 0–360 degrees | uniform PDF on open interval (0, 360) |
|  | *dispersal radius* | ( unlimited ) length units | student's *t* distribution with DOF = 1 |
|  |  |  |  |
|  | *"planted" trees (baseline)* | 2000 trees year^-1^ | calculated from "undisturbed" scenario |
|  | | | |
| • | denotes parameter range / value after *Nathan et al.* [10]. | | |

*Section 1: Initial (ecological) conditions*

This is a function called within the main model (see Section 3, below) that guarantees each model run begins with trees in the same initial positions. (Subsequent landscape disturbance conditions are generated in Section 2.) These initial conditions for the model are based on the key model parameters shown in Table S1.

**%% Begin routine:**

%

function ASSTMIG_IC_function(Nts, DOF)

%

% Authors: Eli Lazarus & Brian McGill

% Created: April 2011

% Amended: October 2012, December 2012

%

% Permissions: OPEN -- but please cite: Lazarus & McGill, "Pushing the pace

% of tree species migration" (PLOS ONE, 2014).

%

% Start migration-front model with the same initial conditions each time.

%

% Takes 'number of time steps' (Nts) and 'DOF' (degrees of freedom) from

% main function.

rand('seed', 1)

**%% Domain details**

r = 1*10^2; % rows

c = 2*10^4; % columns

domain = zeros(r, c);

[col, row] = meshgrid(1:c, 1:r); % col, row based on c & r, determined above

**%% Ecological parameters**

% Fecundity

MA = 450; % max age -- see 'longevity' below

seeds = 300 + round(2.*(1:MA).^2); % number of seeds as function of age

% year at which a tree matures and begins generating seed crops:

matgen = round(3 + 57.*rand(size(domain)));

% seed-crop interval:

tintgen = round(1 + 7.*rand(size(domain)));

longevity = round(18.*gamrnd(5, 1, r, c)); % Gamma distribution of mortality

**%% Storage**

tree = zeros(size(domain));

age = zeros(size(domain));

tint = NaN(size(domain));

tsr = NaN(size(domain));

store_active = zeros(1, Nts);

store_mass = zeros(r, Nts);

**%% Initial conditions -- using trnd (Matlab t-distribution function)**

for TEE = 1:100 % based on 100 cycles

for ff = 1:r

filler = ceil(abs(trnd(ones(1, 10000)))); % all given same IC

Zs1 = find(filler > c);

Zs2 = find(filler <= 0);

filler(Zs1) = [ ];

filler(Zs2) = [ ];

tree(ff, filler) = 1;

age(ff, filler) = round(3 + 397.*rand(1, length(filler)));

tint(ff, filler) = tintgen(ff, filler);

tsr(ff, filler) = round(1 + 7.*rand(1, length(filler)));

end

end

die = find(age >= longevity); % make sure domain has no ages over max age

tree(die) = 0;

age(die) = 0;

tsr(die) = 0;

% reset the "dead" trees with parameters within bounds

tree(die) = 1;

mark = find(tree == 1);

age(mark) = age(mark) + 1;

tint(mark) = tintgen(mark);

tsr(mark) = tsr(mark) + 1;

mature = find(age == matgen);

tint(mature) = tintgen(mature);

tsr(mature) = 0;

**%% Save domain**

save(['ASSTMIG_IC_DOF' num2str(DOF) '.mat']);

**%% End routine**

*Section 2: Hurst-exponent landscape*

This code builds the normalized Hurst-exponent landscape used to determine disturbance patterns: *H* = 0.99 generates a highly autocorrelated landscape; *H* = 0 generates a random (spatially uncorrelated) landscape. The '.mat' file saved at the end of this routine is called by the main model. Positions of trees in the initial conditions are checked against disturbed areas; trees in a disturbed patch are removed from the domain.

**%% Begin routine**

%

**%% Hurst-exponent landscape**

%

% Authors: Brian McGill & Eli Lazarus

% Created: April 2011

% Amended: October 2012, December 2012

%

% Permissions: OPEN -- but please cite: Lazarus & McGill, "Pushing the pace

% of tree species migration" (PLOS ONE, 2014).

%

% After Hastings & Sugihara (1993), "Fractals: A user's guide for the natural

% sciences", New York: Oxford University Press, p. 76.

clear all

rand('seed', 0)

**%% Choose Hurst exponent**

H = 0.99; % highly autocorrelated landscape

% H = 0; % uncorrelated landscape

% Dimensions here should match row, column dimensions of main model (see Section 3)

rowin = 100; % rows

colin = 20000; % columns

bRand = 1;

% temporary domain adjustment...

rowt = rowin - 1;

colt = colin - 1;

row = colt;

col = rowt;

**%% Build landscape**

phase = 2*pi*rand(row + 1, col + 1);

phase = phase';

[I, J] = meshgrid(0:row, 0:col);

ampl = sqrt(I.^(1 - 2*H).*J.^(1 - 2*H)); % deterministic phase

ampl(1, :) = 0;

ampl(:, 1) = 0;

if(bRand)

ampl = abs(randn(col + 1, row + 1)).*ampl; % randomize amplitude

end

areal = cos(phase).*ampl;

aimag = sin(phase).*ampl;

spectrum = areal + i*aimag;

d = real(ifft2(spectrum)); % landscape output

**%% Normalize landscape**

% Values between 0 and 1

d = d + abs(min(min(d)));

d = d./(max(max(d)));

**%% Routine to identify where percentage thresholds are (for % disturbance)**

dx = 1;

for T = 0.01:0.001:1

p = length(find(d <= T))/numel(d);

store_p(idx, 1) = p;

idx = idx + 1;

end

%%

figure

plot(0.01:0.001:1, store_p, '.k-', 'MarkerSize', 10)

grid on

%% Save domain

save('H99_IC.mat')

**%% End routine**

*Section 3: Migration model*

This code comprises the main routine of the tree migration model, and calls on the code shown above in Sections 1 and 2.

**%% Begin routine**

%

**%% Tree-migration model**

%

% Authors: Eli Lazarus & Brian McGill

% Created: April 2011

% Amended: October 2012, December 2012

%

% Permissions: OPEN -- but please cite: Lazarus & McGill, "Pushing the pace

% of tree species migration" (PLOS ONE, 2014).

%

% Note that this routine calls:

%

% 'ASSTMIG_IC_function' -- which takes as input variables 'Nts'

% (number of time steps) and 'DOF' (degrees of freedom). See Section 1.

%

% A Hurst-exponent landscape (here, denoted 'H0_IC.mat' or 'H99_IC.mat').

% See Section 2.

%%

clear all

close all

**%% Domain variables**

r = 1*10^2; rows

c = 2*10^4; columns

% degrees of freedom for dispersal kernel

for DOF = [1 2 3 5 10 30 100]

% K = multiplier (in %) for assisted migration; set K = 0 for no assistance

for K = 0:0.1:2

% thresholds for landscape disturbance (based on 'store_p' in Section 2)

for turb = [0.369 0.413 0.444 0.472 0.497 0.523 0.550 0.582 0.626]

Nts = 500; % years (number of time steps)

DOF = 1; % degrees of freedom for t distribution

% builds initial conditions -- see Section 1...

ASSTMIG_IC_function(Nts, DOF)

load(['ASSTMIG_IC_DOF' num2str(DOF) '.mat'])

if(0) % OFF if other Hurst landscape is ON

% Hurst landscape = random (not autocorrelated)

load('H0_IC.mat', 'D');

HUMANS = zeros(size(D));

HUMANS(D < turb) = 0;

HUMANS(D > turb) = 1;

tree(HUMANS == 0) = 0;

age(HUMANS == 0) = 0;

tsr(HUMANS == 0) = 0;

end

if(1) % OFF if other Hurst landscape is ON

% Hurst landscape = autocorrelated

load('H99_IC.mat', 'd');

HUMANS = zeros(size(d));

HUMANS(d < turb) = 0;

HUMANS(d > turb) = 1;

tree(HUMANS == 0) = 0;

age(HUMANS == 0) = 0;

tsr(HUMANS == 0) = 0;

end

rand('seed', 1) % ensures same starting point in random-number generator

ALL_tree = zeros(size(domain));

tstore_lings = 0;

tstore_lost = 0;

**%% Begin routine...**

for year = 1:Nts; % for each year...

if mod(year, 10) == 0

disp(year)

end

die = find(age >= longevity); % old trees die

% make "dead" tree cells available again

tree(die) = 0;

age(die) = 0;

tsr(die) = 0;

if year == 1

treeo = tree; % save the IC

end

tree_old = tree;

mark = find(tree == 1);

% Update the ages, crop interval & time since seed

% release clocks...

age(mark) = age(mark) + 1;

tint(mark) = tintgen(mark);

tsr(mark) = tsr(mark) + 1;

% for young trees...

mature = find(age == matgen); % ...once a tree is mature...

tint(mature) = tintgen(mature); % ...it's given a seed-crop interval

tsr(mature) = 0; % and its "time since last release" is set to zero.

**%% Identify the trees producing seeds this year...**

% which trees are in their seed-crop interval year?

active = find(tsr == tint);

if ~isempty(active)

% track how many active trees there are this year

store_active(year) = length(active);

end

for idx = 1:length(active) % steps through each "active" tree

xa = row(active(idx)); % identify the row...

ya = col(active(idx)); % and column of given "active" tree

surv_rate = 0.00001 + 0.01999.*rand; % seedling survival

% number of seedlings (must be an integer)

lings = round(surv_rate*seeds(age(xa, ya)));

% tracks total seedlings in model

tstore_lings = tstore_lings + lings;

if lings >= 1

for g = 1:length(lings) % for each seedling (per active tree)

clear thisx thisy

radius = ceil(abs(trnd(DOF))); % pick a disersal distance

theta = 360*rand; % pick a compass direction (in degrees)

% find the cell where that seedling goes...

if theta > 0 && theta < 90

tcol = round(radius*cosd(theta));

trow = round(radius*sind(theta));

if trow >= xa

over = mod(trow, xa);

if over == 0

thisx = 1;

thisy = tcol + ya;

else

thisx = 1 + r - over;

thisy = tcol + ya;

end

else

thisx = xa - trow;

thisy = tcol + ya;

end

elseif theta == 90

thisx = xa;

thisy = radius + ya;

elseif theta > 90 && theta < 180

tcol = round(radius*cosd(180 - theta));

trow = round(radius*sind(180 - theta));

if xa + trow >= r

over = mod(trow + xa, r);

if over == 0

thisx = r;

thisy = tcol + ya;

else

thisx = over;

thisy = tcol + ya;

end

else

thisx = xa + trow;

thisy = tcol + ya;

end

elseif theta == 180

if radius + xa < r

thisx = xa + radius;

elseif radius + xa > r

addon = mod(radius + xa, r);

thisx = addon;

elseif radius + xa == r

thisx = r;

end

thisy = ya;

elseif theta > 180 && theta < 270

tcol = round(radius*sind(theta - 180));

trow = round(radius*cosd(theta - 180));

if xa + trow >= r

over = mod(trow + xa, r);

if over == 0

thisx = r;

else

thisx = over;

end

else

thisx = xa + trow;

end

thisy = ya - tcol;

elseif theta == 270

thisx = xa;

thisy = ya - radius;

elseif theta > 270 && theta < 360

tcol = round(radius*sind(360 - theta));

trow = round(radius*cosd(360 - theta));

if xa > trow

thisx = xa - trow;

elseif trow > xa && trow + xa < r

thisx = r - (trow - xa);

elseif trow > xa && trow + xa >= r

addon = mod(trow - xa, r);

thisx = r - addon;

if addon == 0

thisx = xa;

end

elseif trow == xa

thisx = 1;

end

thisy = ya - tcol;

elseif theta == 360 || theta == 0

if xa > radius

thisx = xa - radius;

elseif radius > xa && radius + xa < r

thisx = r - (radius - xa);

elseif radius > xa && radius + xa >= r

addon = mod(radius - xa, r);

thisx = r - addon;

if addon == 0

thisx = xa;

end

elseif radius == xa

thisx = 1;

end

thisy = ya;

end

if thisy >= 1 && thisy <= c

if tree(thisx, thisy) == 0

if HUMANS(thisx, thisy) == 1

tree(thisx, thisy) = 1;

age(thisx, thisy) = 0;

end

end

end

% counts the trees that leave either end of the

% domain...

if thisy < 1

tstore_lost = tstore_lost + 1;

end

if thisy > c

tstore_lost = tstore_lost + 1;

end

end % closes seeding loop

end % closes 'if seedlings' statement

end % closes "active" trees loop

tsr(active) = 0; % reset tsr clock on active site

tintgen = round(1 + 7.*rand(size(domain))); % generate a new tint

tint(active) = tintgen(active); % just active sites pick new tint

**%% Planting (assisted migration)**

if year >= 200 && K > 0

% new trees per year for ecological baseline

% (DOF = 1; undisturbed domain)

natural_mu_dTT = 2000;

TOPLANT = K*natural_mu_dTT;

sites = find(tree == 0); % find free sites in domain

scramble = randperm(length(sites)); % randomize their sequence

sites = sites(scramble);

plant = sites(1:TOPLANT);

tree(plant) = 1;

age(plant) = 0;

end

**%% Identify the migration front**

% metric based on mean position per row of domain...

for hhh = 1:r

pickm = find(tree(hhh, :) == 1);

if ~isempty(pickm)

store_mass(hhh, year) = mean(pickm);

else

store_mass(hhh, year) = 0;

end

end

store_lings(1, year) = tstore_lings;

store_lost(1, year) = tstore_lost;

for xx = 1:r % step through all the rows...

% how many open spaces are there?

blanks(xx, year) = length(find(ALL_tree(xx, 1:edge) == 0));

end

newts = tree - tree_old; % number of new trees?

if sum(sum(tree)) == 0 % if there are no trees left, stop run.

break

end

end % closes time (year) loop

% Seedling mortality (actual):

LING_MORT = store_lost./store_lings;

mu_blanks = mean(blanks, 1);

dmblanks = diff(mu_blanks);

mu_mr = 10*mean(dmblanks(200:end));

% final storage variables (USE THESE for migration front plots)

massfront = mean(store_mass, 1);

dmf = diff(massfront);

mean_dmf = 10*mean(dmf(60:end)); % in meters, b/c cell size is 10 m

**%% Save the workspace**

save(['AM_HUM_DOF1_K' num2str(K*100) '_H99_dist' num2str(turb*1000) '.mat']);

end % closes 'disturbance' loop

end % closes 'K' loop

end % closes DOF loop

**%% End routine**

*Section 4: Routine for figures*

This code generates the basic formats of the figures published in this article.

**%% Begin routine**

%% Figures for tree migration model

%

% Authors: Eli Lazarus & Brian McGill

% Created: April 2011

% Amended: October 2012, December 2012, February 2013

%

% Permissions: OPEN -- but please cite: Lazarus & McGill, "Pushing the pace

% of tree species migration" (PLOS ONE, 2014).

%

% This routine generates the basic forms of the published figures.

% Note that the data file names ('.mat') called are those specified in the main model.

%%

clear all

close all

%% Fig. 2 (DOF plot)

idx = 1;

for i = [1 2 3 5 10 30 100]

load(['AM_ECOL_DOF' num2str(i) '.mat'], 'massfront', 'dmf');

MF_set(:, idx) = massfront;

DMF_set(:, idx) = dmf;

DOF_set(idx) = mean(dmf(200:end));

DOF_set_max(idx) = max(dmf(200:end));

DOF_set_min(idx) = min(dmf(200:end));

DOF_set_std(idx) = std(dmf(200:end));

idx = idx + 1;

end

dofs = [1 2 3 5 10 30 100];

figure

hold on

plot(dofs, 10.*DOF_set, '.k-', 'LineWidth', 2, 'MarkerSize', 15)

plot(dofs, 10.*DOF_set + 10.*DOF_set_std, '.b-')

plot(dofs, 10.*DOF_set - 10.*DOF_set_std, '.c-')

hold off

xlabel('DOF')

ylabel('Migration rate')

%% Fig. 3 (Fragmentation effects given H = 0.99 & H = 0)

% for Hurst == 0

idx = 1;

for i = 10:10:90

load(['AM_HUM_DOF1_K0_H0_dist' num2str(i) '.mat'], 'massfront', 'dmf');

MFh_set_H0(:, idx) = massfront;

DMFh_set_H0(:, idx) = dmf;

muh_set_H0(idx) = mean(dmf(200:end));

maxh_set_H0(idx) = max(dmf(200:end));

minh_set_H0(idx) = min(dmf(200:end));

stdh_set_H0(idx) = std(dmf(200:end));

idx = idx + 1;

end

% for Hurst = 99

idx = 1;

for i = 10:10:90

load(['AM_HUM_DOF1_K0_H99_dist' num2str(i) '.mat'], 'massfront', 'dmf');

MFh_set_H99(:, idx) = massfront;

DMFh_set_H99(:, idx) = dmf;

muh_set_H99(idx) = mean(dmf(200:end));

maxh_set_H99(idx) = max(dmf(200:end));

minh_set_H99(idx) = min(dmf(200:end));

stdh_set_H99(idx) = std(dmf(200:end));

idx = idx + 1;

end

figure

hold on

plot(10:10:90, 10.*muh_set_H0, 'sk-', 'MarkerSize', 10)

plot(10:10:90, 10.*muh_set_H0 + 10.*stdh_set_H0, '.b-')

plot(10:10:90, 10.*muh_set_H0 - 10.*stdh_set_H0, '.b-')

plot(10:10:90, 10.*muh_set_H99, 'ok-', 'MarkerSize', 10)

plot(10:10:90, 10.*muh_set_H99 + 10.*stdh_set_H99, '.c-')

plot(10:10:90, 10.*muh_set_H99 - 10.*stdh_set_H99, '.c-')

plot(0, 10.*DOF_set(1), '.k', 'MarkerSize', 15)

plot(0, 10.*DOF_set(1) + 10.*DOF_set_std(1), '.b-')

plot(0, 10.*DOF_set(1) - 10.*DOF_set_std(1), '.b-')

refline([0 0])

hold off

xlabel('Percent disturbed (d)')

ylabel('Migration rate')

ylim([-150 150])

%% Fig. 4 (diminishing change in mean migration rate)

idx = 1;

for i = 10:10:200

load(['AM_ECOL_DOF1_K' num2str(i) '.mat'], 'massfront', 'dmf');

MFK_set(:, idx) = massfront;

DMFK_set(:, idx) = dmf;

muDMFK(idx) = mean(dmf(201:300));

maxDMFK(idx) = max(dmf(201:300));

minDMFK(idx) = min(dmf(201:300));

stdDMFK(idx) = std(dmf(201:300));

idx = idx + 1;

end

% Fig. 4A

figure

hold on

plot(10:10:200, 10.*muDMFK, '.k-', 'MarkerSize', 15)

plot(10:10:200, 10.*muDMFK + 10.*stdDMFK, '.b-')

plot(10:10:200, 10.*muDMFK - 10.*stdDMFK, '.b-')

plot(0, 10.*DOF_set(1), 'ok', 'MarkerSize', 10)

plot(0, 10.*DOF_set(1) + 10.*DOF_set_std(1), '.b-')

plot(0, 10.*DOF_set(1) - 10.*DOF_set_std(1), '.b-')

hold off

xlabel('K')

ylabel('Mean migration rate')

% diminishing returns...

temp_mudmfk = zeros(1, 21);

temp_mudmfk(1, 1) = DOF_set(1);

temp_mudmfk(1, 2:end) = muDMFK;

dfmuDMFK = diff(temp_mudmfk);

% Fig. 4B

figure

plot(10:10:200, 10.*dfmuDMFK, '.k-', 'MarkerSize', 15)

xlabel('K')

ylabel('Change in mean migration rate (in m)')

%% Fig. S2 (saturation of the migration front)

figure

hold on

plot(MFK_set, 'b')

plot(MFK_set(:, [5 10 15]), 'b', 'LineWidth', 2)

plot(MFK_set(:, end), 'r', 'LineWidth', 2)

plot(MF_set(:, 1), 'k', 'LineWidth', 2)

hold off

grid on

xlabel('Time (years)')

ylabel('Mass Front (position in m)')

%% Fig. 5 (assisted migration rates under different disturbance scenarios)

idx = 1;

for i = 20:20:200

idx2 = 1;

for j = [369 413 444 472 497 523 550 582 626]

load(['AM_HUM_DOF1_K' num2str(i) '_H99_dist' num2str(j) '.mat'], 'massfront', 'dmf');

muDMFK_dist(idx2, idx) = mean(dmf(201:300));

stdDMFK_dist(idx2, idx) = std(dmf(201:300));

idx2 = idx2 + 1;

end

idx = idx + 1;

end

figure

hold on

plot(20:20:200, 10.*muDMFK_dist(1, :), '.k-')

plot(20:20:200, 10.*muDMFK_dist(2, :), '.b-')

plot(20:20:200, 10.*muDMFK_dist(3, :), '.c-')

plot(20:20:200, 10.*muDMFK_dist(4, :), '.g-')

plot(20:20:200, 10.*muDMFK_dist(5, :), '.y-', 'LineWidth', 2)

plot(20:20:200, 10.*muDMFK_dist(6, :), 'k-')

plot(20:20:200, 10.*muDMFK_dist(7, :), 'b-')

plot(20:20:200, 10.*muDMFK_dist(8, :), 'c-')

plot(20:20:200, 10.*muDMFK_dist(9, :), 'r-')

plot(10:10:200, 10.*muDMFK, 'b-', 'LineWidth', 2)

plot(0, 10.*DOF_set(1), 'ob')

hold off

**%% End routine**

**Figure Captions (supplementary information)**

**Figure S1.** Schematics of our tree migration model: (A) representative initial condition without disturbance (arrows indicate how the periodic boundary operates on seed dispersal); final landscapes (B) without disturbance and (C) with disturbance; (D) sequence in a model year in which (D2) new trees are added by natural dispersal and (D3) by assisted colonization, resulting in (D4) the final landscape as shown. Actual model outputs are shown in Fig. 1.

**Figure S2.** Plot of mean front position versus time for increasing levels of assisted migration in the absence of landscape disturbance. Black line shows the natural, background migration rate (~130 m yr^-1^), given a *t*-distribution dispersal kernel with DOF = 1. In this baseline scenario, approximately 2000 new trees grow in the domain per year. Blue lines show the effects of assisted migration regimes in which an additional *K*% trees per year are "planted", for *K* = 10–200%. Bold blue lines denote 50% increments; *K* = 200% is shown in red.
